# Prednisolone Targets Claudins in Mouse Brain Blood Vessels

**DOI:** 10.3390/ijms25010276

**Published:** 2023-12-24

**Authors:** Alexander G. Markov, Anastasia E. Bikmurzina, Arina A. Fedorova, Ekaterina P. Vinogradova, Natalia M. Kruglova, Igor I. Krivoi, Salah Amasheh

**Affiliations:** 1 Department of General Physiology, St. Petersburg State University, 199034 St. Petersburg, Russia; a.markov@spbu.ru (A.G.M.); nayaspb16@gmail.com (A.E.B.); a.fedorova@spbu.ru (A.A.F.); n.kruglova@spbu.ru (N.M.K.); iikrivoi@gmail.com (I.I.K.); 2 Interoception Laboratory, Pavlov Institute of Physiology RAS, 199034 St. Petersburg, Russia; 3 Department of Higher Nervous Activity and Psychophysiology, St. Petersburg State University, 199034 St. Petersburg, Russia; e.vinogradova@spbu.ru; 4 Institute of Veterinary Physiology, Freie Universität Berlin, 14163 Berlin, Germany

**Keywords:** blood–brain barrier, tight junctions, claudins, prednisolone, behavior

## Abstract

Endothelial cells in brain capillaries are crucial for the function of the blood–brain barrier (BBB), and members of the tight junction protein family of claudins are regarded to be primarily responsible for barrier properties. Thus, the analysis of bioactive substances that can affect the BBB’s permeability is of great importance and may be useful for the development of new therapeutic strategies for brain pathologies. In our study, we tested the hypothesis that the application of the glucocorticoid prednisolone affects the murine blood–brain barrier in vivo. Isolated brain tissue of control and prednisolone-injected mice was examined by employing immunoblotting and confocal laser scanning immunofluorescence microscopy, and the physiological and behavioral effects were analyzed. The control tissue samples revealed the expression of barrier-forming tight junction proteins claudin-1, -3, and -5 and of the paracellular cation and water-channel-forming protein claudin-2. Prednisolone administration for 7 days at doses of 70 mg/kg caused physiological and behavioral effects and downregulated claudin-1 and -3 and the channel-forming claudin-2 without altering their localization in cerebral blood vessels. Changes in the expression of these claudins might have effects on the ionic and acid–base balance in brain tissue, suggesting the relevance of our findings for therapeutic options in disorders such as cerebral edema and psychiatric failure.

## 1. Introduction

The blood–brain barrier (BBB) is a complex of cell structures and physiological mechanisms interacting with each other and controlling the transport of substances between blood and the central nervous system in order to maintain the conditions that are optimal for brain function. The BBB includes astrocytes’ end feet, pericytes, brain capillary endothelial cells (BCECs) connected by a tight junction (TJ), and basal membranes. Endothelial TJs are the most important structural component in the BBB, and molecular alterations in the expression of some TJ proteins are crucial in determining alterations in the control of the BBB’s vascular permeability [1,2,3].

A superfamily of transmembrane proteins, claudins, is the main component of the TJ, and they determine the selectivity of paracellular transport for molecules, ions, and water. The extracellular loops of claudins of neighboring endothelial cells interact with each other over the intercellular clefts. A different ratio of claudins in TJs is a prerequisite for changing tight junctional properties and the junctions’ tightness. Based on these functional characteristics, claudins are classified into two large groups: channel formers, e.g., claudin-2, and barrier formers, e.g., claudin-1, -3, and -5 [4]. Taking the dominant idea of the BBB’s impermeability into account, the main emphasis in the study of the molecular determinants of the barrier properties of the brain’s blood vessel endothelium was on claudin-3 and -5, which reduced the permeability of the tissue barrier [5,6,7,8]. However, the study of mRNA in mouse BCECs showed the presence of transcripts not only for tightening claudins (-1, -3, -5), but also for the channel-forming protein claudin-2 [9]. Nevertheless, it can be assumed that a change in the ionic composition of brain tissue fluid during nerve cell activity requires the inclusion of all mechanisms to maintain the acid–base balance, including the paracellular diffusion of ions and water. In addition, vasogenic edema results from the compromise of the BBB, resulting in the net influx of water and proteinaceous fluid into the interstitium [10]. Claudin-2 is an important component of the TJ and provides a selective paracellular channel for small cations and water [11,12,13]. There are no data about claudin-2 at the protein level in brain tissue so far.

Interest in the mosaic of claudins expressed in cerebral vessels has also been raised by recent data on their role in the development of psychiatric disorders. These studies focus on the contributions of claudin-5 as the main molecular component of the BBB [14,15]. Under stress, the TNFα/NFκB signaling pathway is involved in a change in claudin-5 [15]. Nevertheless, the positive correlations found for claudin-12 and ZO-1 mRNA transcripts, together with the altered characteristics of psychiatric disorders [14], suggest the involvement of other claudins in this process.

The detection of bioactive substances—especially endogenous ones—that are capable of altering the expression of TJ proteins and affecting the barrier’s permeability is one of the primary challenges in understanding the regulation of the BBB. In this regard, the ability of glucocorticoids to affect the BBB’s permeability, which has been demonstrated in cell lines, has come into focus [16,17,18,19]. However, it should be kept in mind that mice and humans share a similar and complex TJ profile in vivo, but this complexity is widely lost under in vitro conditions [20]. Thus, in order to reveal the role of glucocorticoids in the regulation of the BBB’s function, in vivo experiments are essential. Data on glucocorticoids’ effects on the expression of the TJ proteins in adult animals are scarce. Antenatal treatment with dexamethasone reduced the expression of claudin-5 in the brains of pups [21]. In addition, it is important to study endogenous compounds circulating in the blood and preventing functional disorders in injury [22]. Preliminary administration of dexamethasone reduced the negative effect of lipopolysaccharide on the epithelial barrier properties in rat ileum [23]. Glucocorticoid administration was able to reduce brain edema in patients with intracranial tumors [24]. The commonly applied prednisolone is a 1,2-dehydrated analog of the endogenous glucocorticoid cortisol. This modification of the structure causes an approximately tenfold increase in selectivity for the glucocorticoid receptor compared to the mineralocorticoid receptor.

Knowledge of molecular mechanisms behind the regulatory effects of glucocorticoids and their functional significance for brain pathology could be useful for the development of a new and effective therapeutic strategy. The main goals of this study were to test the presence of the channel-forming protein claudin-2 in addition to tightening claudins in mouse brain blood vessels and to test the ability of an exogenous glucocorticoid, prednisolone, to modulate the expression of claudins in this tissue. In addition, systemic and behavioral changes were examined with prednisone treatment.

## 2. Results

### 2.1. Physiological Effects of Prednisolone Administration

**Figure 1:**
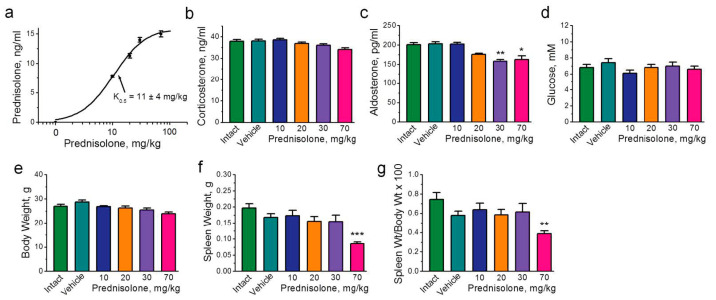
Physiological effects of exposure to prednisolone. Mice were injected intramuscularly for 7 days with different doses (mg/kg) of prednisolone, as indicated. Intact mice were not injected. Control mice were injected with vehicle (0.9% NaCl). (**a**) Dependence of the level of prednisolone in the blood serum on the dose of prednisolone administered. The solid curve was fitted with the Hill equation; the calculated constant K0.5 for doses of prednisolone is indicated. (**b**–**d**) Evaluation of the level of corticosterone (**b**), aldosterone (**c**), and glucose (**d**) in the blood serum. (**e**–**g**) The effects of exposure to prednisolone on body weight (**e**), spleen weight (**f**), and the ratio of spleen weight to body weight (**g**). *n* = 5–16. One-way ANOVA. * *p* < 0.05, ** *p* < 0.01, and *** *p* < 0.001 compared with the corresponding control (vehicle-treated group).

Intramuscular injections of prednisolone (10 to 70 mg/kg) in a dose-dependent manner increased serum prednisolone levels with a constant K_0.5_ = 11 ± 4 mg/kg (Figure 1a). Prednisolone is a synthetic glucocorticoid; therefore, the most sensitive criterion for its action is the change in the level of endogenous corticoids. Moreover, as these may influence electrolyte balance and glucose metabolism, glucose and body weight, as well as spleen weight, were measured. The serum corticosterone levels showed only a slight downward trend with the increase in the prednisolone dose (Figure 1b). However, the level of aldosterone decreased at doses of prednisolone of 30 to 70 mg/kg (Figure 1c). Injections of prednisolone did not significantly affect the glucose levels (Figure 1d). Although no effect on body weight was observed (Figure 1e), 70 mg/kg of prednisolone (*p* < 0.001) reduced the spleen weight (Figure 1f); the ratio of the spleen weight to body weight also decreased (*p* < 0.01; Figure 1g). Such effects are characteristic of glucocorticoids [25].

**Figure 2:**
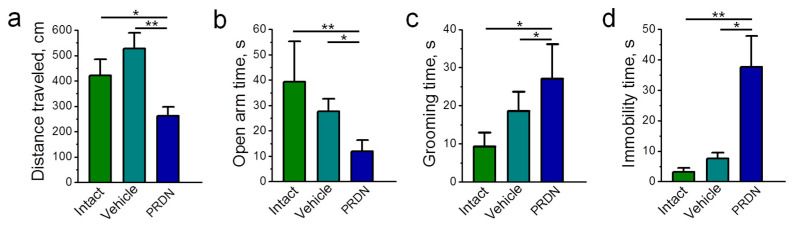
Behavioral effects of exposure to prednisolone (PRDN). A variety of behavioral domains were studied, and this included tests for assessing locomotor activity, anxiety-like behavior, and depression-related behavior. Mice were injected intraperitoneally for 7 days with prednisolone at a dose of 70 mg/kg. Control mice were injected with vehicle (0.9% NaCl). (**a**) Distance traveled during a 5 min period. (**b**) Time spent with open arms measured in an elevated plus-maze in a 5 min test. (**c**) Grooming duration in an elevated plus-maze in a 5 min test. (**d**) Depression-related behavior assessed in the Porsolt forced swim test. *n* = 5–13. One-way ANOVA. * *p* < 0.05 and ** *p* < 0.01—compared as indicated by the horizontal bars.

The behavioral changes in the conditions of intraperitoneal administration of prednisolone (70 mg/kg) for 7 days (Figure 2) were also characteristic of the systemic action of glucocorticoids [26,27,28]. Although the subsequent molecular analyses focused on approaches with intramuscular injection, this subset was chosen to exclude any peripheral muscular effects during the behavioral analyses. These analyses included locomotor activity, as indicated by distance traveled (Figure 2a), anxiety-like behavior (open arm time, grooming time; Figure 2b,c), and depression-related behavior (immobility time; Figure 2d). All behavioral observations revealed significant changes in accordance with the effects of the application (vehicle) and prednisolone, respectively.

### 2.2. Prednisolone Administration Modulated Claudin Expression in the Mouse Brain

**Figure 3:**
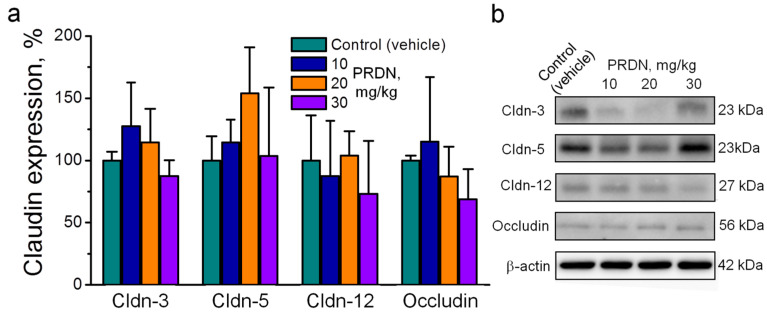
Effects of exposure to prednisolone (PRDN) on the expression of claudin (Cldn) and occludin in the mouse brain. Mice were injected intramuscularly for 7 days with different doses (mg/kg) of prednisolone, as indicated. Control mice were injected with vehicle (0.9% NaCl). (**a**) Averaged Western blot measurements. *n* = 4–9. One-way ANOVA. (**b**) Corresponding representative Western blots. No significant differences were found in comparison with the control (vehicle-treated group). Samples derived from the same experiment and the blots were processed in parallel.

We first examined whether exposure to relatively low doses of prednisolone could already modulate claudin expression. Although some dispersion of values could be observed, intramuscular injections of prednisolone at doses of 10, 20, and 30 mg/kg did not significantly affect the expression of the major TJ proteins that were widely expressed in the vascular endothelium, namely, claudin-3, -5, and -12 and occludin (Figure 3). Notably, treatment with prednisolone at a dose of 20 mg/kg caused a tendency to increase the expression of claudin-5. This could be important given the emphasis on the study of claudin-5 in the development of psychological disorders [14,15]. However, this change was not significant (*p* = 0.29).

**Figure 4:**
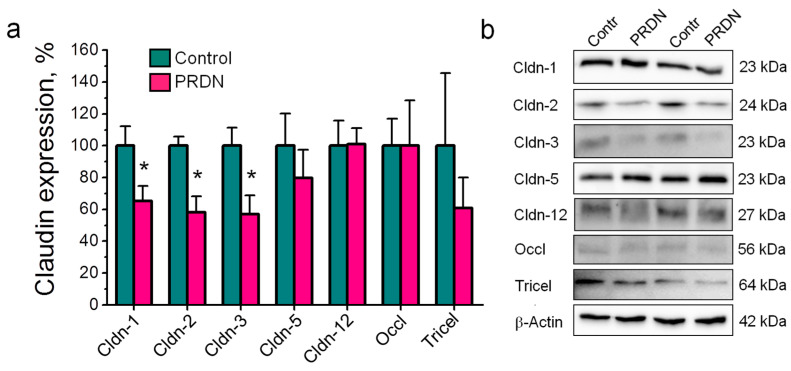
Effects of exposure to prednisolone (PRDN) on the expression of claudin (Cldn), occludin (Occl), and tricellulin (Tricel) in the mouse brain detected via immunoblotting. Mice were injected intramuscularly for 7 days with prednisolone at a dose of 70 mg/kg. Control mice were injected with vehicle (0.9% NaCl). (**a**) Densitometric analysis. *n* = 4–16. One-way ANOVA. * *p* < 0.05 compared to the controls. (**b**) Corresponding representative Western blots. Samples derived from the same experiment and the blots were processed in parallel.

Next, we tested the effects of 70 mg/kg prednisolone on the expression of an even broader spectrum of claudins, which additionally included claudin-1 and -2. In these experiments, injections of prednisolone reduced the expression of claudin-1, -2, and -3; the expression of claudin-5 and -12, occludin, and tricellulin did not change (Figure 4).

### 2.3. Prednisolone Administration Did Not Alter Claudin Localization in Cerebral Blood Vessels

**Figure 5:**
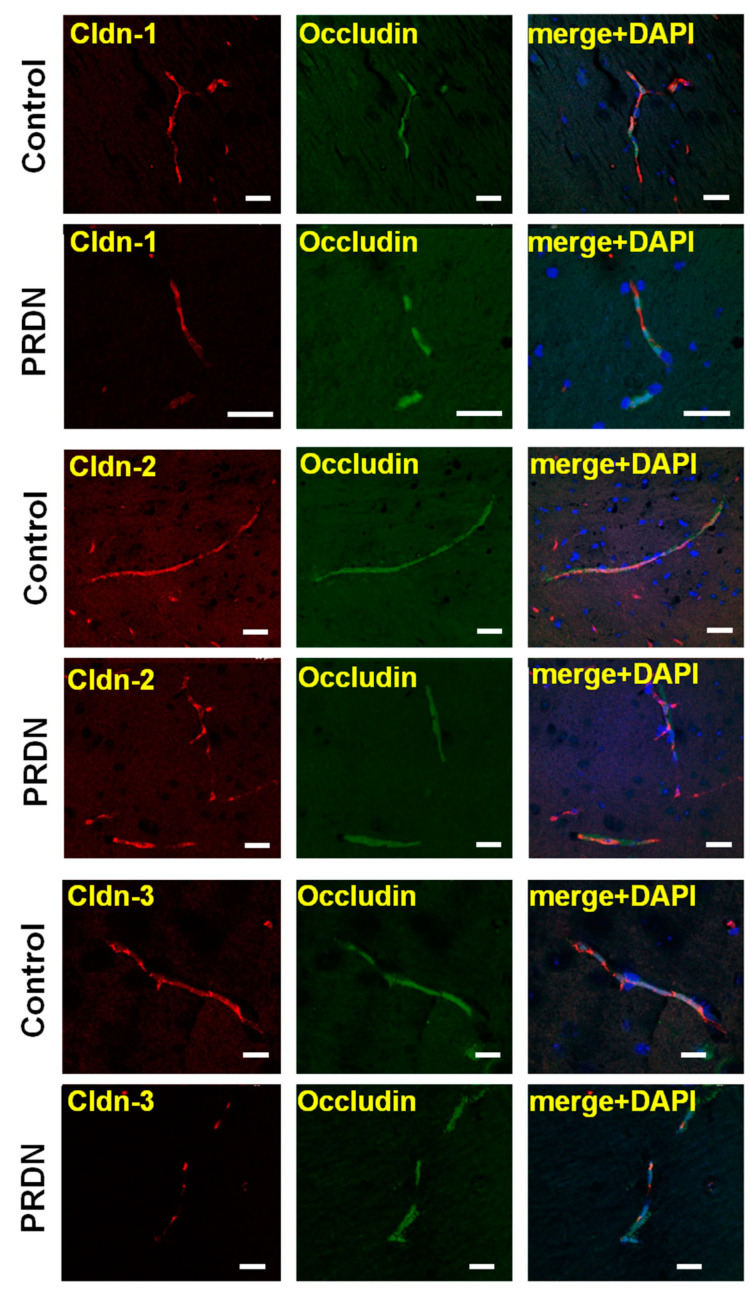
Exposure to prednisolone (PRDN) did not alter the localization of claudin (Cldn) in cerebral blood vessels. Mice were injected intramuscularly for 7 days with prednisolone at a dose of 70 mg/kg. Control mice were injected with vehicle (0.9% NaCl). The distribution of claudins and occludin and the oval shape of the nuclei of endothelial cells (DAPI) coincided with the characteristic arrangement of blood vessels in the brain (scale bars: 20 μm).

Immunofluorescent staining was used to determine the localization of claudins in the brain tissue. The specific signals corresponding to claudin-1, -2, -3, -5, and -12 and occludin were detected in both the control and prednisolone-treated groups. The extended features of fluorescent signal distribution and the oval shape of the nuclei indicated that the signal was located in the region of vascular endothelial cells. Although the expression of claudin-1, -2, and -3 was reduced by the action of prednisolone (Figure 4), as expected, their localization was not affected (Figure 5).

## 3. Discussion

The blood vessels of the brain are a key structure providing selective transport and barrier properties between the blood plasma and the extracellular space. These functions are determined by a mix of carriers and channels of the plasma membrane of endothelial cells, as well as by a mosaic of endothelial TJ proteins—primarily claudins [2,3]. While the expression of tightening claudins in the BBB’s tissues is well documented, the presence of channel-forming claudins is still unclear. A difference in the spectrum of claudins in cell culture and brain tissue [20] is also a problem that needs to be addressed. Thus, the in vivo identification of the spectrum of claudins in brain vessels, as well as their modulators of an exogenous and endogenous nature, is one of the prioritized tasks in the study of the BBB.

In this study, we tested the presence of the channel-forming protein claudin-2 in addition to tightening claudins in the blood vessels of the frontal lobes of the mouse brain and tested the ability of an exogenous glucocorticoid, prednisolone, to modulate claudin expression in this tissue. The novelty of our findings is the following:

(1) In addition to claudin-1, -3, -5, and -12, the expression of the channel-former claudin-2 in the vascular endothelium of the mouse brain was first shown at the protein level.
(2) Prednisolone downregulated the channel-former claudin-2 and the tightening claudins claudin-1 and -3 without changes in the expression of claudin-5 and -12. These changes were accompanied by deviations in depression-related behavior.
(3) Prednisolone modulated claudin expression without changes in their localization in cerebral blood vessels.

Our analysis showed the presence of all claudins (1, 2, 3, 5, and 12) in the vascular endothelium of the brain of mice, the transcripts of which were previously determined in the vascular endothelium [9]. The molecular diversity of claudins that differed in their properties emphasizes that the barrier properties of the endothelium of the blood vessels of the brain are variable, and this allows the search for new agents that change these properties. 

The main result of the analysis was the determination of channel-forming claudin-2 in the endothelium of the cerebral vessels. This fact allows us to look at the molecular basis of the barrier properties of the cerebral vascular endothelium in a completely new way. The expression of claudin-2 has been reported to mediate paracellular permeability for small cations such as Na^+^, as well as for water [11,29]. Therefore, our new finding suggests that there are molecular determinants in the structure of the TJ that are involved in modulating the balance of water and salt in the brain. Vasogenic edema occurs when blood vessels, including TJ structures, are damaged [30]. Furthermore, disruption of ionic homeostasis in the brain plays an important role in the occurrence of pathological conditions of the brain, particularly in cerebral edema [30,31]. Although these studies did not focus on the claudin-dependent barrier properties of the blood–brain barrier, doses reaching at least up to 100 mg /kg were commonly used in experiments, surpassing those employed in our study, in accordance with the benefit–risk ratio of our experimental approach [32,33]. However, our findings regarding the change in claudin-2 expression may further add an important missing aspect, as this protein, due to its paracellular pore function [11], can also be regarded as relevant for ionic and acid–base balance in the brain. However, if and to which extent the analyzed claudins might regulate ionic and acid–base balance in brain tissue in vivo remains to be elucidated.

Claudin-12 was recently identified in the vessels of the mouse brain [20]. The confirmation of the expression of claudin-12 in our experiments emphasizes the need for a wide range of claudins for the barrier properties of the vascular endothelium.

The presence of claudin-1 in the BBB seems to be variable and has not been definitively clarified. Claudin-1 was found in immortalized a micro-vascular endothelial cell line of the human brain [16], primary microvascular endothelial cells of the rat brain [34], and primary endothelial cells of the mouse brain [35], but it was absent in another immortalized brain microvascular endothelial cell line from the cerebral cortex of neonatal mice [36]. Our data confirm that claudin-1 is part of the endothelial barrier in the brain vessels. As claudin-1 is known to be a barrier-forming TJ protein [37], one might assume that it tightens the TJ of the BBB. 

As the molecular basis of the barrier properties of the BBB, focus is often laid on claudin-3 and -5. Claudin-3, which tightens the TJ [38], is present in the various vascular endothelial cell lines of the brain [39,40], as well as in the brain tissue of mice [5,8]. Its participation in the formation of the BBB is not questioned. The selective loss of claudin-3 from TJs of the BBB in experimental autoimmune encephalomyelitis and human glioblastoma multiforme led to the conclusion that it might be a central component for the determination of the BBB’s integrity in vivo [5]. Claudin-5 is a basic, constant molecular component of the BBB. It is present in all vascular endothelial cell lines [16,18,33,34,35,36,39,40,41,42], as well as in the vascular endothelium of the brain tissue [5,7,8,43]. The functional role of claudin-5 in the formation of barrier properties was demonstrated in experiments with claudin-5-deficient mice. These animals had high permeability of the BBB for solutes with a size of up to 800 Da [6]. Temporal knockout of the *claudin-5* gene resulted in similar changes in the barrier function and in behavioral changes in tests with thyroliberin treatment. Restoration of claudin-5 expression led to the withdrawal of the observed changes [44]. Thus, in blood vessels of the mouse brain, endotheliocyte TJs are formed by claudins that are opposite in their properties, which should ensure the adaptation of paracellular transport when the conditions for the function of the nervous tissue change.

In addition to claudins, other proteins are also known to be localized in the TJ. Here, we showed tricellulin to be present in the endothelium of the brain vessels, which also expands the understanding of the barrier properties of the cerebral vascular endothelium. Occludin and tricellulin are involved in the regulation of the paracellular passage of macromolecules [45,46,47,48]. 

The general effect of glucocorticoids in tissues is manifested in a decrease in the permeability of the epithelium and the restoration of the barrier properties of the tissue barrier during injury [23,49]. Glucocorticoids have a similar effect on the BBB and are used in the clinic for the treatment of cerebral edema [30]. In our experiments, an effect of prednisolone on the expression of TJ proteins in the endothelium of cerebral vessels was found. A change in the molecular level of claudins occurred with a dose of 70 mg/kg; this was associated with a reduction in the concentration of aldosterone in the blood plasma, a decrease in spleen weight, and a change in behavior.

Studies of glucocorticoid effects on the cerebral vascular endothelial cell line unambiguously demonstrated an increase in transendothelial resistance and a decrease in permeability for macromolecules of a small mass [16,18,36,41,50]. It has been shown in different cerebral vascular endothelial cell lines that glucocorticoids cause an increase in the expression of claudin-5 [36,41] and occludin [16,18,36,41]. The level of claudin-1 did not change with hydrocortisone treatment [16]. In our experiments, however, prednisolone caused a decrease in the level of tightening claudin-1 and -3, as well as in that of channel-forming claudin-2. Thus, it is worth noting the possible difference in the action of glucorticoids on vascular endothelial cell lines and in the blood vessels of the mouse brain [20]. The solution of the question of the species-specificity of changes in the expression of claudins in the endothelium of blood vessels in the brain of mice is promising for subsequent experiments.

Recent studies demonstrated the association between BBB disruption based on changes in the TJ proteins of the cerebral vascular endothelium and the development of psychiatric disorders, with claudin-5 and -12 being considered as the main targets [14,15]. Glucocorticoid treatment is known to induce depressive behavior in mice [26,27,28]. In this study, the administration of prednisolone caused decreased locomotor activity and anxiety-like and depression-like behavior; intriguingly, this occurred without changes in the expression of claudin-5 and -12. This observation suggests the possible involvement of other TJ proteins in the development of psychiatric disorders. Whether it could be claudin-1, -2, or -3 modulated by prednisolone in this study is not yet clear. Furthermore, other possible consequences of changes in the TJ on brain function under prednisolone treatment should be mentioned. The entry of leukocytes and other immune cells across the BBB into the CNS is an early event in inflammatory disorders of the CNS [51,52]. Barrier dysfunction can contribute to neurological disorders in a passive way through the vascular leakage of blood-borne molecules into the CNS and in an active way by guiding the migration of inflammatory cells into the CNS. Both ways may directly be linked to alterations in molecular composition and the function of TJ proteins [53,54]. Thus, as blood–brain barrier disruption is known to promote inflammation by enabling leukocytes, T cells, and other immune cells to migrate via the paracellular routes across the blood–brain barrier and to infiltrate the parenchyma of the CNS, the change in claudin levels through prednisolone treatment can also be considered as a promising therapeutic strategy for preventing neuroinflammation.

In our study, only male mice were used to exclude effects mediated by estrogens, which might have further effects related to TJ proteins, as, e.g., claudin-5 is known to be affected by estrogen [55]. Therefore, our study does not exclude the possibility of different effects in female mice. Generally, further research on the molecular mechanisms of the integrity, modulation, and rearrangement of the BBB in various types of brain disorders is required.

## 4. Materials and Methods

### 4.1. Animals

All procedures were performed in accordance with established practices, as described in the National Institutes of Health Guide for Care and Use of Laboratory Animals [56].

Male laboratory mice (age: 2–3 months, weight: 25–30 g) were purchased from “Rappolovo” (NRC “Kurchatov Institute”, St. Petersburg, Russia). Animals were held in a temperature- and humidity-controlled room at the animal facility of St. Petersburg University (Russia). They were exposed to a 12-h dark/light interval and ingested water and food (Lufa-ITL GmbH, Kiel, Germany) ad libitum.

Mice were intramuscularly injected with vehicle (0.9% NaCl) or prednisolone at 10, 20, 30, or 70 mg/kg body weight once per day for 7 days. One day after the last injection (1:00 p.m.), the animals were sacrificed via decapitation, and the brain was removed. The frontal lobes were separated, frozen in liquid nitrogen, and then stored at −80 °C for later Western blot or immunohistochemistry assays. The weights of the animal and the spleen were measured using Sartorius-1602 MP (Göttingen, Germany).

After sampling, the blood was centrifuged for 15 min at 800× *g* (Multi Spin MSC-3000, Riga, Latvja) and stored at −80 °C. Hormone levels were determined by using ELISA (DRG^®^ Aldosterone ELISA, DRG; Corticosterone rat/mouse ELISA and EIAab^®^ Human Prednisolone ELISA; St. Louis, MO, USA) with sensitivity thresholds of 4.2 pg/mL for aldosterone, 4.1 ng/mL for corticosterone, and 0.312 ng/mL for prednisolone. The procedure followed was in accordance with the standard protocols of the respective kits.

The serum glucose level was measured with an electronic blood glucose meter (Accu-Chek Active, Roche Diabetes Care GmbH, Mannheim, Germany).

### 4.2. Behavioral Experiments

In the behavioral experiments, mice were intraperitoneally injected with prednisolone at a dose of 70 mg/kg for 7 days. Behavioral testing was performed between 1:00 p.m. and 7:00 p.m. After each test, the mice were returned to their home cages, and all apparatuses were cleaned with 70% ethanol to prevent bias based on olfactory cues.

An elevated plus-maze test was conducted to assess anxiety-like behavior [57]. The elevated plus-maze consisted of two open arms (30 cm length × 5 cm width) with 3 mm high ledges and two closed arms (30 cm × 5 cm) with 15 cm high transparent walls (Open Science, Moscow, Russia). The floors of the arms and the central square (5 cm × 5 cm) were made of white plastic plates and were elevated to a height of 55 cm above the floor. Arms of the same type were arranged on opposite sides of the maze. The center of the maze was illuminated at 100 lux. Each mouse was placed at the center of the maze facing one of the open arms and was recorded for 5 min. The distance traveled (cm), number of total entries into the arms, and the time spent in the open arms were measured for 5 min.

The Porsolt forced swim test [58] was performed to assess depression-related behavior. A Plexiglas cylinder (30 cm height × 10 cm diameter) was placed in a test chamber (Open Science, Moscow, Russia). A video camera was mounted on the ceiling of the test chamber and positioned directly above the cylinder. The mice were placed in the cylinder, which was filled with water (approximately 22 °C) to a height of 10 cm. Immobility time was recorded over a 6 min test period.

### 4.3. Western Blotting

Tissues were mechanically homogenized in ice-cold lysis buffer (1 M Tris-Cl pH 7,4, 1 M MgCl_2_, 0.5 M EDTA, 0.5 M EGTA, protease inhibitors). Supernatants from a short centrifugation (1000× *g*, 5 min, 4 °C) were centrifuged (42,100× *g*, 30 min, 4 °C), and the resulting pellets containing the membrane protein fraction were resolved in lysis buffer. Protein contents were determined using the BCA protein assay reagent (Pierce, Rockford, IL, USA) quantified with a plate reader (Tecan, Grodig, Austria). Samples were mixed with SDS buffer Laemmli (Sigma, St. Louis, MO, USA), loaded on a 12.5% SDS polyacrylamide gel, and electrophoresed. Proteins were assessed by immunoblotting while employing rabbit anti-claudin-1, -2, -12 (1:1000), -3, -5 (1:2000), anti-occludin (1:2000), and anti-tricellulin (1:3000) primary antibodies, as well as β-actin (1:10,000). To detect bound antibodies, peroxidase-conjugated goat anti-rabbit IgG or goat anti-mouse IgG antibodies and the Lumi-LightPLUS chemiluminescence detection system and a Western blotting kit (Roche, Mannheim, Germany) were used. Signals were visualized through luminescence imaging (Fusion FX7, Vilber Lourmat, Eberhardzell, Germany). For comparison of the Western blot signals, densitometry analysis was performed using quantification software (Multi Gauge V2.3, FujiFilm, Tokyo, Japan). Detected proteins were normalized using β-actin as the loading control.

The following antibodies were used: rabbit anti-claudin-1 (#ab56417, 1:50, Abcam, Cambridge, UK), rabbit anti-claudin-2 (#51-6100, Invitrogen, Carlsbad, CA, USA), rabbit anti-claudin-3 (#34-1700, Invitrogen, Carlsbad, CA, USA), rabbit anti-claudin-5 (#34-1600, Invitrogen, Carlsbad, CA, USA), rabbit anti-claudin-12 (#38-8200, Invitrogen, Carlsbad, CA, USA), rabbit anti-occludin (#33-1500, Invitrogen, Carlsbad, CA, USA), rabbit anti-tricellulin (#700191, Life Technologies, Carlsbad, CA, USA), goat anti-rabbit IgG (#7074, Cell Signaling Technologies, Leiden, The Netherlands), goat anti-mouse IgG (#7076, Cell Signaling Technologies, Leiden, The Netherlands), and mouse anti-β-actin (Sigma-Aldrich, Taufkirchen, Germany).

### 4.4. Immunohistochemistry

Tissues were fixed in 4% paraformaldehyde for 48 h, treated according to standard histological tissue processing, and embedded in paraffin. For immunostaining, paraffin was removed from the cross-sections (8 μm) using a xylol–ethanol gradient. For antigen retrieval, sections were boiled in 1 mM EDTA buffer solution. To block non-specific binding sites, the sections were incubated in PBS containing 6% (vol/vol) goat serum and 1% BSA (blocking solution) for 60 min at room temperature. All subsequent washing procedures were performed with this blocking solution. Tissues were incubated at room temperature for 60 min in rabbit anti-claudin-1, -2, -3, -5, -12 (1:100), anti-occludin (1:200), and anti-tricellulin (1:500) primary antibodies in blocking solution and, after two washes, further incubated with Alexa Fluor goat anti-mouse IgG and Alexa Fluor goat anti-rabbit IgG (Molecular Probes, Eugene, OR, USA) diluted to 1:500 in blocking solution for 45 min at room temperature. Furthermore, nuclei were stained with DAPI (Roche, 1:1000). Sections were mounted with ProTags MountFluor (Biocyc, Luckenwalde, Germany). Fluorescence images were obtained with a confocal laser scanning microscope (Leica TCS SP5, Wetzlar, Germany).

### 4.5. Materials

Chemicals were purchased from Sigma-Aldrich, unless stated otherwise.

### 4.6. Statistics

Data are given as the mean ± SEM. The statistical significance of the differences between means was evaluated using one-way ANOVA. Statistical analysis was performed using the GraphPad Prism 8 software (GraphPad; San Diego, CA, USA). A probability value of *p* < 0.05 was considered statistically significant.

## 5. Conclusions

In conclusion, the channel former claudin-2, the ambiguously reported protein claudin-12, the barrier formers claudin-1, -3, and -5, occludin, and tricellulin are localized in TJs of the endothelium of mouse brain vessels. They are involved in the regulation of the paracellular passage of ions and solutes through the endothelium, and selective changes in their expression can be assumed to result in changes in the permeability of the BBB. Our finding that prednisolone administration downregulates the barrier formers claudin-1 and -3 and the channel former claudin-2 could be useful for the development of novel and effective therapeutic strategies for brain pathologies. Considering the ability of glucocorticoids to modulate Na,K-ATPase [59], as well as the existence of a functional interaction between Na,K-ATPase and claudins in vivo [22], further studies of this mechanism appear promising.

## Data Availability

The data that support the findings of this study are available from the corresponding author upon reasonable request.
